# High-Level Vibration for Single-Frequency and Multi-Frequency Excitation in Macro-Composite Piezoelectric (MFC) Energy Harvesters, Nonlinearity, and Higher Harmonics

**DOI:** 10.3390/mi14010001

**Published:** 2022-12-20

**Authors:** Majid Khazaee

**Affiliations:** 1 Department of AAU Energy, Aalborg University, Pontopidanstraede 111, 9220 Aalborg, Denmark; mad@energy.aau.dk; 2 School of Biomedical Engineering, Faculty of Engineering, The University of Sydney, NSW 2000, Australia

**Keywords:** piezoelectric, real-time vibration, random signal, white noise, nonlinearity

## Abstract

This paper presents an extensive experimental investigation to identify the influence of signal parameters on a piezoelectric harvester’s performance. A macro-fibre composite energy harvester was studied as an advanced, flexible, high-performance energy material. Gaussian white noise, and single-frequency and multi-frequency excitation were used to investigate nonlinearity and multiple-frequency interactions. Using single low and high frequencies, we identified the nonlinearity of the harvester’s vibration. Multi-frequency excitation with a series of low-to-high-frequency harmonics mimicked the practical vibration signal. Under such multi-frequency excitation, the harvester’s nonlinear behaviour was studied. Finally, the interaction effects among multiple frequencies were identified. The results show that under pure resonant excitation, high-level vibration led to high-level mechanical strain, which caused nonlinear vibration behaviour. Moreover, it was shown that the different harmonics excited the various structure bending modes, which caused the nonlinearity of multi-frequency excitation. The first four harmonics of the real-time signal were important. The experimental results emphasise the resonant nonlinearity and interactions of multi-frequency excitation effects.

## 1. Introduction

Piezoelectric materials with electrical-to-mechanical conversion abilities are alternatives to power electronic devices, as they utilise the often-wasted electrical energy. Piezoelectric Vibration Energy Harvesting (PVEH) has found industrial [1] and bio-related [2] applications. By moving toward self-powered electronics with PVEH, electronic devices and biomedical sensors/actuators can be installed in inaccessible areas, such as in vivo or remote areas, using the often-wasted available energy [3].

Practical vibration signals from machines or the environment are often random, multi-frequency signals. Despite extensive PVEH from practical vibrations [4,5], a laboratory-controlled sinusoidal input is typically used as an excitation signal, which is different from real-time vibration systems, as real-time vibration signals are stochastic. Real-time randomness may affect the efficacy of piezoelectric harvester optimisation practices [6], emphasising the importance of multi-frequency vibration analysis of piezoelectric energy harvesters (PEHs). Some studies have analysed PEHs using random vibration methods, such as white noise [7] and Gaussian-coloured noise [8]. Moreover, PVEH using wind force, as another familiar environmental energy source, entails randomness, which has been experimentally investigated [9]. These studies often focus on the PEH output without properly focusing on different excitation frequencies and multi-modal beam models. In PVEH, the frequency and amplitude of the vibration source are prominent parameters of the output power of a PEH [10,11]. The PEH maximum voltage is a vibration signal with an excitation frequency close to the natural frequency [12]. This process is called frequency matching [13,14], and many studies have presented good frequency matching [15] or broadband frequency matching [16] techniques. The vibration amplitude is another vital element that impacts the output power. According to the typical linear models, PEH power depends on the square of the amplitude of the external sinusoidal vibration [17,18].

Mechanical strain is directly linked to the electrical charge flow in a piezoelectric material. In other words, piezoelectric strain corresponds to electrical energy generation. Previous studies have pointed to the nonlinearity of PEH performance under high-level single-frequency resonant excitation [19]. The nonlinear effect is more significant when the beam is more flexible due to specific boundary conditions [20]. In the clamped–free beam, the most used PVEH configuration, the strain is non-uniform, and the clamped-end region has the maximum strain over the whole volume [16]. Thus, the piezoelectric beam experiences large strain in this configuration. A high strain level, especially under resonant clamped–free excitation, causes a nonlinear effect on the PEH; however, previous studies have not presented a comprehensive frequency-spectrum analysis of high-level vibrations.

PVEH is a multidisciplinary research area closely connected to vibration characteristics. Yet, many vibrational phenomena, such as nonlinearity and multi-frequencies, should be addressed. Modelling literature studies typically use single-frequency and linear assumptions [3,21]. In contrast, there is a lack of practical and nonlinear vibration signal analysis. The unmet goals include comprehensively analysing PEHs subjected to multi-frequency vibration signals, the interaction effects of different frequencies, the signal vibration randomness, and high-level vibrations. Such comprehensive analysis would provide an accurate energy estimation of the practical vibration sources and assess the current linear methods regarding real-time vibration signals. The present study introduces a deep experimental work on vibration signals with randomness and multi-frequencies. The effects of increasing the vibration level of different single-frequency and multi-frequency signals were studied. Moreover, the interaction effects of the different vibration modes were analysed. These innovative investigations significantly enrich the PVEH knowledge of modelling and output power estimation.

The work of this manuscript is categorised as follows: Section 2 discusses the need for multi-frequency analysis with a real-time vibration signal demonstration. A linear modelling technique is presented in Section 3 for natural frequency estimation. Moreover, the model for natural frequency estimation was validated with experimental tests, as shown in Section 4. Section 5 deals with the results and discussion, including the randomness effect, the effects of increasing the vibration level of single-frequency and multi-frequency vibrations, and the interaction effects of multi-modal vibrations on a PEH. Section 6 presents the concluding remarks and the proposed future works. The comparisons of high-level vibrations of single-frequency and multi-frequency vibration signals show the high-strain-level nonlinearity of the PEH. In addition, the interaction effects among different vibration modes can be significant.

## 2. The Need for Multi-Frequency Harmonic Analysis

**Figure 1:**
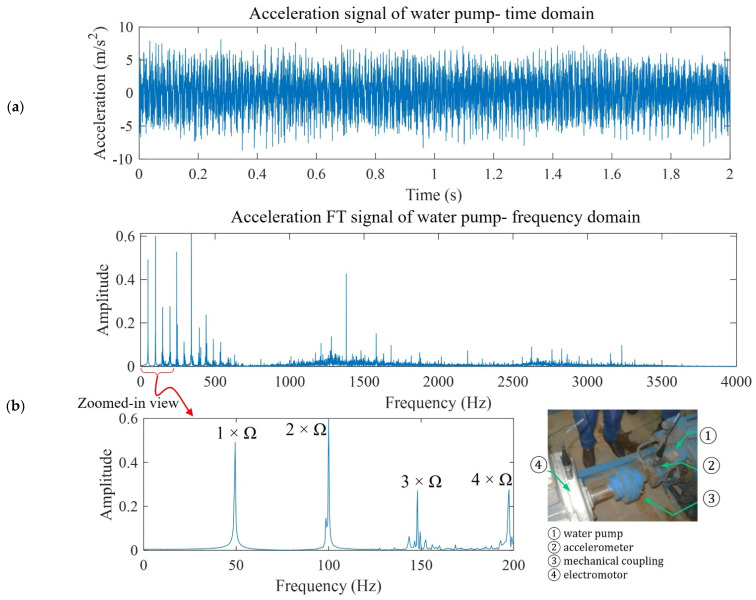
Real example of vibration from an operation machine: (**a**) acceleration and (**b**) frequency spectra of vibration signal from a water pump during operation.

Practical vibration sources are random vibration signals comprising a series of harmonic signals. The Fourier Transform (FT) of a time-domain vibration signal demonstrates the frequency information of the vibration source. Therefore, a full-range frequency analysis is required for energy harvesting power estimation. As a practical example, the water pump acceleration signal of a running water pump with rotation speed Ω = 2970 rpm (Ω ≈ 49.5 Hz) is shown in Figure 1a [22]. This acceleration signal is influenced by the roller bearing elements and structural elements, which affect the FT peak amplitudes due to factors such as bearing ball diameter and number of balls [23,24]. This acceleration signal is not purely harmonic and consists of harmonic multipliers of Ω, according to the FT signal shown in Figure 1b. In the zoomed-in view of the FT signal, the four dominant frequencies are shown and marked with 1 × Ω, 2 × Ω, 3 × Ω, and 4 × Ω.

A common assumption in piezoelectric harvester analysis is the single-mode assumption, which simplifies modelling by only considering a frequency range around the fundamental harvester frequency. Analytical modelling studies are often simplified for single-frequency harmonic vibrations, but practical vibration signals are not single-frequency harmonics. High-frequency harmonics may excite a harvester’s high-vibration modes. Since an electrode covers the whole piezoelectric surface, electrical charge cancellation may be experienced in multi-vibration modes. Therefore, a full-range frequency analysis is needed to assess the high-frequency harmonics and interaction effects among high frequencies.

Moreover, most analytical models present a linear model for energy harvesting power estimation. The linear model assumes a linear relationship between input acceleration and output voltage. Nevertheless, assessing linearity under conditions of multiple vibration frequencies is difficult due to different vibration mode shapes.

## 3. Closed-Form Solutions for Mechanical and Electrical Responses

$a_{n}$ are the sampled acceleration signal measurements of acceleration function a(t). To obtain the closed-form solutions under this general load, the acceleration is represented by a series of harmonic functions using the Fast Fourier Transform (FFT).

(1) $$A_{i}=\overset{N-1}{\underset{n=0}{\sum }}a_{n}e^{-j\;\frac{2\pi in}{N}}$$

where $A_{i}$ is the external acceleration FFT and N is the number of sampled measurement data.

For the vibration solution of the harvester, the piezoelectric beam deformation is denoted by

(2) $$w\left(x,t\right)=\overset{\infty }{\underset{r=1}{\sum }}\phi_{r}\left(x\right)\;\eta_{r}\left(t\right)$$

where $\phi_{r}\left(x\right)$ is the displacement-dependent function (beam mode shapes) and $\eta_{r}\left(t\right)$ is the time-dependent mechanical deformation function.

**Figure 2:**
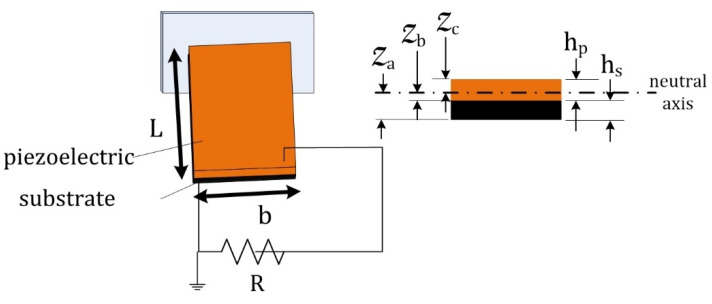
Piezoelectric sample with dimensions.

The electromechanical equations of a piezoelectric unimorph without tip mass are given by [15]

(3) $${\ddot{\eta }}_{r}\left(t\right)+2\zeta_{r}\omega_{r}{\dot{\eta }}_{r}\left(t\right)+\omega_{r}^{2}\eta_{r}\left(t\right)+\Upsilon_{r}V_{R}\left(t\right)=\sigma_{r}\;a_{n}$$

(4) $$C_{P}{\dot{V}}_{R}\left(t\right)+\left(1/R\right)V_{R}\left(t\right)-\Lambda_{r}{\dot{\eta }}_{r}\left(t\right)=0$$

where the parameters are given in Table 1. The unimorph parameters are shown in Figure 2.

Assuming a linear framework, mechanical displacement $\eta_{r}\left(t\right)$ and output voltage $V_{R}\left(t\right)$ are the summation of the outputs from each harmonic $\Omega_{i}$, starting from i=0 to +∞. Individual harmonic components are denoted with ${\bar{\eta }}_{r,\;\Omega_{i}}$ and ${\bar{V}}_{R,\Omega_{i}}$, respectively. Therefore, the overall mechanical vibration and electrical responses are

(5.a) $$\eta_{r}\left(t\right)≅\Delta \Omega \overset{N-1}{\underset{i=0}{\sum }}{\bar{\eta }}_{r,\;\Omega_{i}}.e^{j\Omega_{i}t}$$

(5.b) $$V_{R}\left(t\right)≅\Delta \Omega \overset{N-1}{\underset{i=0}{\sum }}{\bar{V}}_{R,\Omega_{i}}.e^{j\Omega_{i}t}$$

where ${\bar{\eta }}_{r,\;\Omega_{i}}$ is the mechanical response and ${\bar{V}}_{R,\Omega_{i}}$ is the piezoelectric voltage response due to a nominal excitation harmonic with $\Omega_{i}$ frequency. Note that the over-bar indicates the magnitude.

For obtaining ${\bar{\eta }}_{r,\;\Omega_{i}}$ and ${\bar{V}}_{R,\Omega_{i}}$, the harmonic solution analysis of the piezoelectric energy harvester differential is carried out [25], as the steady-state relationships can be given by

(6.a) $$\left(\omega_{r}^{2}-\Omega_{i}^{2}+j2\zeta_{r}\omega_{r}\Omega_{i}\right){\bar{\eta }}_{r,\;\Omega_{i}}+\gamma_{r}{\bar{V}}_{R,\Omega_{i}}=\sigma_{r}\;A_{i}$$

(6.b) $$\left(\frac{1}{R}+jC_{P}\Omega_{i}\right){\bar{V}}_{R,\Omega_{i}}=\overset{\infty }{\underset{r=1}{\sum }}j\Omega_{i}\Lambda_{r}\;{\bar{\eta }}_{r,\;\Omega_{i}}$$

Eliminating mechanical response ${\bar{\eta }}_{r,\;\Omega_{i}}$ between Equations (6.a) and (6.b), the output voltage can be expressed as

(7) $${\bar{V}}_{R,\Omega_{i}}=A_{i}\left|\frac{\frac{1}{m^{*}}j\Omega_{i}\sum_{r=1}^{\infty }\Lambda_{r}\sigma_{r}\frac{1}{\omega_{r}^{2}-\Omega_{i}^{2}+j2\zeta_{r}\omega_{r}\Omega_{i}}}{\frac{1}{R}+jC_{P}\Omega_{i}+j\Omega_{i}\sum_{r=1}^{\infty }\frac{\Lambda_{r}\gamma_{r}}{\omega_{r}^{2}-\Omega_{i}^{2}+j2\zeta_{r}\omega_{r}\Omega_{i}}}\right|$$

Finally, the total voltage generation is calculated as

(8) $${\bar{V}}_{R}≅\Delta \Omega \left|\overset{N-1}{\underset{i=0}{\sum }}{\bar{V}}_{R,\Omega_{i}}\right|=\Delta \Omega \left|\overset{N-1}{\underset{i=0}{\sum }}A_{i}\frac{\frac{1}{m^{*}}j\Omega_{i}\sum_{r=1}^{\infty }\Lambda_{r}\sigma_{r}\frac{1}{\omega_{r}^{2}-\Omega_{i}^{2}+j2\zeta_{r}\omega_{r}\Omega_{i}}}{\frac{1}{R}+jC_{P}\Omega_{i}+j\omega_{i}\sum_{r=1}^{\infty }\frac{\Lambda_{r}\gamma_{r}}{\omega_{r}^{2}-\Omega_{i}^{2}+j2\zeta_{r}\omega_{r}\Omega_{i}}}\right|$$

The output power is calculated as

(9) $${\bar{P}}_{R}=\frac{{\bar{V}}_{R}{}^{2}}{R}≅\frac{{\Delta \Omega }^{2}}{R}{\left(\left|\overset{N-1}{\underset{i=0}{\sum }}A_{i}\frac{\frac{1}{m^{*}}j\Omega_{i}\sum_{r=1}^{\infty }\Lambda_{r}\sigma_{r}\frac{1}{\omega_{r}^{2}-\Omega_{i}^{2}+j2\zeta_{r}\omega_{r}\Omega_{i}}}{\frac{1}{R}+jC_{P}\Omega_{i}+j\omega_{i}\sum_{r=1}^{\infty }\frac{\Lambda_{r}\gamma_{r}}{\omega_{r}^{2}-\Omega_{i}^{2}+j2\zeta_{r}\omega_{r}\Omega_{i}}}\right|\right)}^{2}$$

This power conversion term is frequency and load dependent, in addition to being dependent on the material and geometrical properties.

Equation (9) contains essential information:

(1) The total output power is calculated using two series over the frequency range and the modal mode shapes for general input acceleration. Therefore, parameters such as $\Upsilon_{r}$ and $\Lambda_{r}$ are mode-shape dependent, in addition to being dependent on the external frequency, which is $\Omega_{i}$. The interaction between the external excitation frequency and the mode shapes is a complex research object, specifically when an electrode covers the piezoelectric layer.
(2) Moreover, there is a linear relationship between the output power and the external square acceleration amplitude. This linear dependency over different frequencies needs to be researched, since excitation and modal mode interactions can be nonlinear.

This paper tackles the above two research questions.

## 4. Experimental Setup and Initial MFC Harvester Characterisation

### 4.1. Experimental Setup

**Figure 3:**
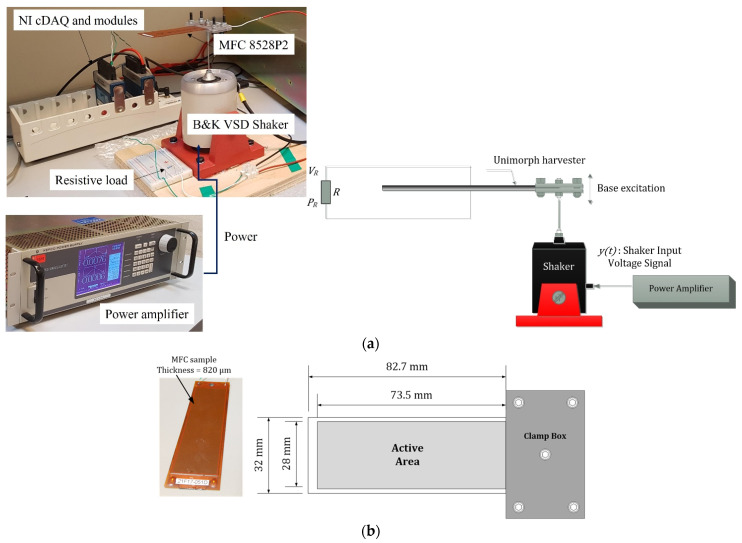
(**a**) Test rig and (**b**) MFC piezoelectric sample dimensions.

Figure 3a shows the test rig for all the experimental measurements. Two aluminium base plates connected a B&K shaker to the piezoelectric sample, tightened with four bolts. The amplifier was controlled with a National Instruments NI 9263 module, which generated analogue voltage signals. A KEPCO BOP 100-10MG amplifier amplified the signals and powered the shaker. The piezoelectric harvester output wires were connected across a resistive load of resistance R. A Data Acquisition (DAQ) system, an 8-channel National Instruments NI 9201 module, was employed for reading the voltage across the resistive load (which was also the voltage across the harvester). The NI modules were placed in an NI cDAQ 9172 chassis connected with a USB cable to the computer.

The shaker input voltage signal, y(t), was varied in frequency and amplitude, and the piezoelectric voltage was measured. The shaker input voltage was a single-frequency or multiple-frequency harmonic.

The piezoelectric sample comprised a macro-fibre composite (MFC) of 0.3 mm in thickness, an aluminium substrate shim of 0.12 mm, and an epoxy rapid 332 bonding layer of ~0.4 mm in thickness. The bonding layer has little effect on the natural frequency, but it is a significant source of damping [26]. Therefore, the bonding layer was neglected in the structural modelling of the natural frequency; however, its effect was considered by extracting the damping coefficient experimentally, so the bonding layer influence was observed. The bonding layer joined the MFC and substrate shim. The MFC was the M-8528-P2 type from Smart Material GmbH (Dresden, Germany) [27], a piezoelectric bending energy harvester. The MFC had seven sub-layers: two Kapton outer layers, two acrylic layers, two electrodes, and one central active piezoelectric layer. More information about the MFC can be obtained from Smart Material Inc. [27]. Figure 3b shows the MFC sample.

### 4.2. PVEH Device Optimum Characterisation

The piezoelectric sample was primarily analysed and characterised with the model and experimental tests presented in this subsection. The material properties for modelling were as follows: Young’s moduli of MFC and aluminium layers were 15.85 [28] and 68.9 GPa, and the corresponding densities were 5540 and 2700 kg/m^3^. The damping coefficient was 5% [29]. The relative permittivity coefficient and piezoelectric constant *d*_31_ were 1800 and −170 × 10^−12^ *C*/*N*, respectively.

For this energy harvester, the first undamped natural frequencies obtained with the current model were compared with those of the experimental tests, as reported in Table 2. The natural frequency difference was 1.3 Hz. The higher-mode natural frequencies are also given in Table 2 and were employed in the multi-modal analysis. This difference in the natural frequencies can be linked to the non-uniform piezoelectric MFC; in practice, the commercial MFC sample had an active area where the piezoelectric material was placed, and on the outer areas, there were only Kapton layers. Including this non-uniformity would have required advanced piezoelectric beam modelling, which was beyond the scope of this paper. For detailed demonstration and modelling of an MFC, one can refer to [16].

**Figure 4:**
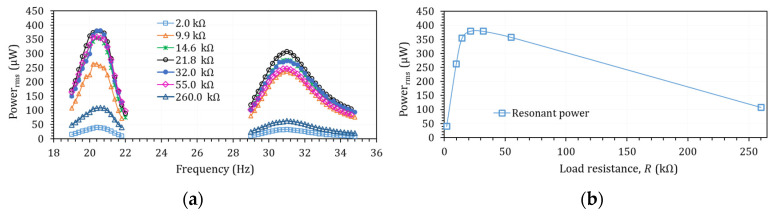
(**a**) Power versus frequency with different load resistances and (**b**) resonant output power for different loads.

Piezoelectric unimorph harvesters are characterised by a natural frequency and optimum power generation. The studied harvester was first evaluated over a frequency range to obtain these conditions. Figure 4a shows power with load resistances over frequency. The resonant frequency was constant for all the load resistances and was equal to $\omega_{r=1}$ = 20.4 Hz. This frequency was the first resonant frequency of the harvester. Moreover, there was another resonant frequency, $\omega_{\mathrm{pc}}$ = 31.0 Hz, associated with the piezoelectric structural effects, here called piezoelectric-coupled frequency. Second, output power versus load resistances were evaluated for optimum load resistance selection. Figure 4b shows output power versus load resistance at the resonant frequency. The optimal load resistance of 21.8 kΩ led to the highest power generation.

## 5. Results and Discussion

The result section provides a comprehensive result set from the experimental tests. Through the experimental tests, power generation with $R_{\mathrm{opt}}$ = 21.8 kΩ was studied with various excitation signals. Note that the load resistance of $R_{\mathrm{opt}}$ = 21.8 kΩ was employed in all experimental results presented in this section. Excitation signals with Added White Gaussian Noise (AWGN), single-frequency harmonics, and multi-frequency harmonics were studied. Moreover, the effect of the vibration level from low-level to high-level vibrations was studied with harmonics with single frequency and multi-frequencies. Because in piezoelectric energy harvesting applications a harvester’s natural frequency is matched to the dominant acceleration frequency, here, $\Omega =\omega_{r=1}$ was assumed.

### 5.1. Effects of Adding White Noise to the Excitation Signal

**Figure 5:**
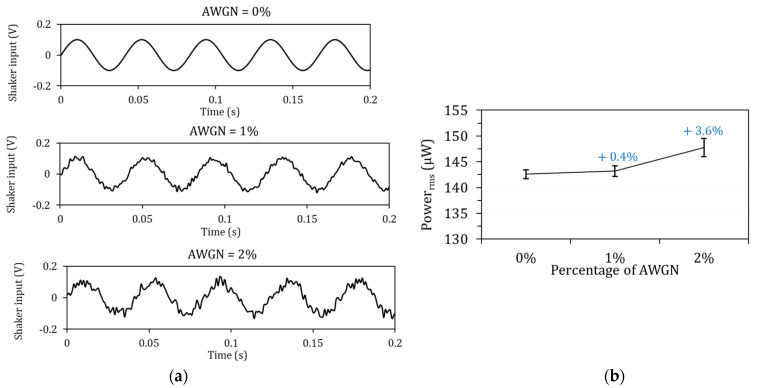
Effects of Added White Gaussian Noise (AWGN) on MFC power generation with fundamental natural frequency excitation: (**a**) shaker vibration signals and (**b**) RMS power with $R_{\mathrm{opt}}$.

Pure analytical solutions often consider a single harmonic signal without noise as an excitation signal. In contrast, practical vibration sources often have added noise. Here, the effect of adding white noise was studied by considering Added White Gaussian Noise (AWGN) for a single harmonic signal with matched frequency, i.e., $\Omega =\omega_{r=1}$. The shaker vibration signal was y(t)=0.1(1+AWGN) sin(2πΩt) (V), where “AWGN” is the white noise percentage, 1% or 2%. The shaker vibration signals with and without AWGN are shown in Figure 5a. The output power results showed that adding white noise increased the power slightly (see Figure 5b). White noise, i.e., random noise with a uniform frequency domain value, caused additional vibration on the piezoelectric harvester, so power generation slightly increased. An important conclusion is that white noise in the practical vibration data did not show a reduction in power influence.

### 5.2. Effects of Increasing the Vibration Level with Single-Frequency Harmonics

A single-frequency harmonic excited the harvester, and the vibration-level effect on the output power was investigated by increasing the harmonic amplitude. This experimental setup tested the linearity assumption of power generation versus input square acceleration amplitude. Note that a single frequency does not imply constant frequency. Various single-frequency harmonics with different frequencies were studied. Considering the matched frequency ($\Omega_{1}=\omega_{r=1}$) up to six harmonics, the natural multiples of the fundamental frequency were studied. Therefore, $\Omega_{i}=i\times \omega_{r=1}$, i=1, …, 6.

Shaker input signals are symbolised with $y_{JK}\left(t\right)=Y_{J}sin\left(K\omega_{r}t\right)$, where Y is the magnitude of the shaker excitation signal and K is the driving frequency multiplier. Subscript J denotes different excitation magnitude levels for one driving frequency. By changing Y, the vibration level moves from low-level to high-level vibrations; in other words, the strain on the piezoelectric material changes from low levels to high levels.

The output power with $R_{\mathrm{opt}}$ was recorded during three independent runs. Table 3 shows the output power, and average and experimental errors for all the harmonics and vibration levels. An experimental error of less than 4% implies experimental repeatability. The sensitivity comparisons showed the dramatic power increase obtained by magnifying the excitation amplitude of all excitation harmonics; however, the trends were not the same for all harmonics. 

**Figure 6:**
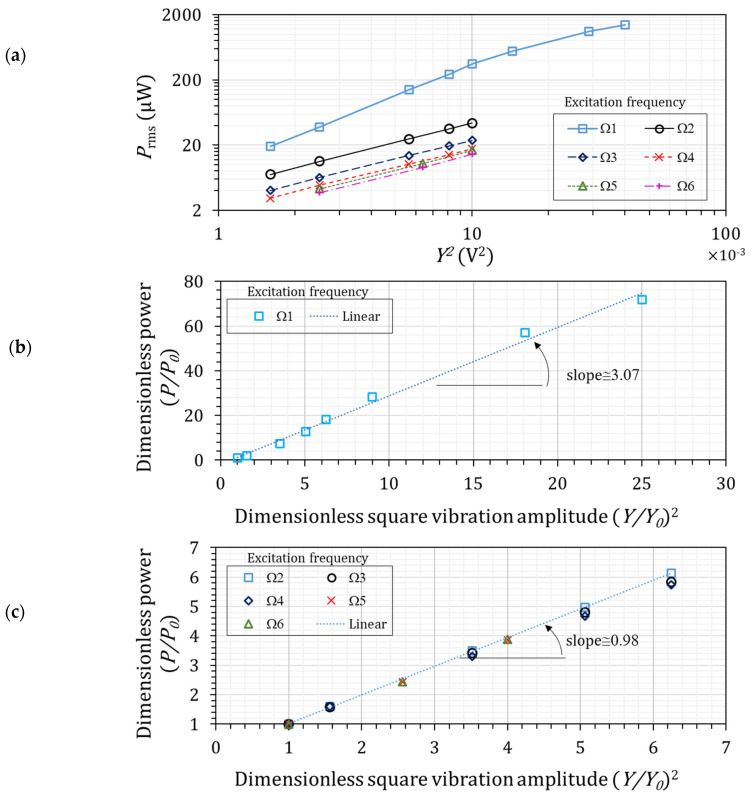
Power variation concerning excitation magnitude in the single-harmonic case with $R_{\mathrm{opt}}$ = 21.8 kΩ. (**a**) plots the output power (P) versus square shaker signal amplitude (Y2) for W1 to W6 frequencies, (**b**,**c**) plot the dimensionless power–vibration values for W1 excitation frequency and for W2 to W6 excitation frequencies respectively.

Output power (P) versus square shaker signal amplitude ($Y^{2}$) for $\Omega_{1}$ to $\Omega_{6}$ frequencies are plotted in Figure 6a. According to the analytical model in Equation (9), the relation between power and square input amplitude is linear for all frequencies; however, Figure 6a demonstrates a separate pattern for $\Omega_{1}$ excitation. This apparent pattern indicates that the resonant excitation frequency differed from the other frequencies.

Further analyses of the power pattern were conducted with the dimensionless study of power versus square vibration amplitude. The dimensionless study divided the parameters into the lowest-level vibration amplitude and power. The dimensionless power–vibration values for $\Omega_{1}$ excitation frequency and for $\Omega_{2}$ to $\Omega_{6}$ excitation frequencies are plotted in Figure 6b and c, respectively.

As shown in Figure 6c, the slope of the fitted line for $\Omega_{2}$ to $\Omega_{6}$ harmonic excitation frequencies was approximately one; power linearly changed by the square of input acceleration at these frequencies. This conclusion is in line with the analytic model in Equation (9). In contrast, for $\Omega_{1}$ frequency, where the excitation frequency was the fundamental natural frequency (Figure 6b), the slope of the fitted line was approximately three.

According to the linear bending theory, the axial strain depends on the beam curvature [15], e.g., $\varepsilon_{xx}\propto \frac{\partial^{2}w}{\partial x^{2}}$. By increasing the vibration level, the strain on a piezoelectric material also increases. Assuming the matched frequency ($\Omega_{1}=\omega_{r=1}$), $\Omega_{1}$-frequency vibration excitation creates resonance deformation on the piezoelectric beam, and the resonance deformation is large. Therefore, a high vibration level is expected to create elevated levels of strain, which goes beyond the linear assumption between the axial strain and beam curvature. However, the experimental dimensionless study showed that for higher harmonics, e.g., $\Omega_{2}$ and beyond, a high vibration level does not cause a high strain level; therefore, the linear analytical model is valid.

It has been demonstrated that nonlinearity exists even in a typical no-added-tip-mass energy harvester. In many typical energy harvesters, an added mass adjusts the fundamental frequency and increases the power [30], inducing physical deformation, i.e., they become noticeably enlarged, at the acting frequency [31]. As observed in Figure 6b, increasing physical deformation creates nonlinearity, and the added-tip-mass effect is expected to enlarge nonlinearity. The tip mass affects the first vibration mode more substantially than other vibration modes (because of the larger first-mode-shape magnitude); therefore, the added-tip-mass effect on the nonlinear effects of resonant excitation is expected to be considerable.

### 5.3. Effects of Increasing the Vibration Level with Multi-Frequency Harmonics

**Figure 7:**
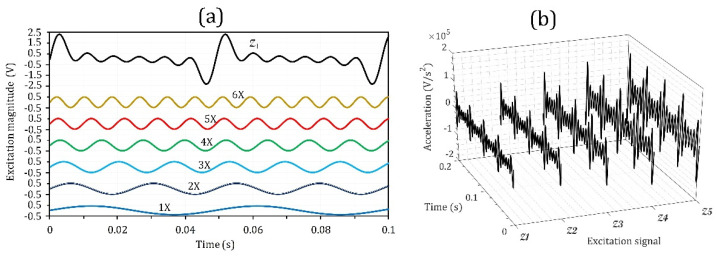
(**a**) Standard multi-harmonic base excitation signal and (**b**) applied acceleration for multi-harmonic base signals.

As demonstrated in Section 2, practical vibration energy sources only have single-frequency signals instead of a series of harmonic multipliers. Thus, a vibration-level study was carried out on a multi-harmonic signal using vibration-level control. The standard baseline excitation signal was the summation of harmonics with $\Omega_{1}$ to $\Omega_{6}$ frequencies, denoted by $Z_{M}\left(t\right)$; it excited the piezoelectric harvester, and the corresponding RMS power with optimum load was $P_{Z_{M}}$. $Z_{M}\left(t\right)$, as given in Equation (10) and shown in Figure 7a.

(10) $$Z_{M}\left(t\right)=M\left[0.04\;\left(V\right)\right]sin\left(\Omega_{1}t\right)+\left[0.05\;\left(V\right)\right]sin\left(\Omega_{2}t\right)+\left[0.05\;\left(V\right)\right]sin\left(\Omega_{3}t\right)+\left[0.05\;\left(V\right)\right]sin\left(\Omega_{4}t\right)\;+\left[0.05\;\left(V\right)\right]sin\left(\Omega_{5}t\right)+\left[0.05\;\left(V\right)\right]sin(\Omega_{6}t)\;$$

where M is the signal magnification factor. Note that $\Omega_{i}=i\times \omega_{r=1}$, i=1, …, 6. In practice, four magnification factors, M = 1, 1.2, 1.5, 1.75, and 2.0, were assigned. The associated acceleration signals from $Z_{1}\left(t\right)$ to $Z_{M=2}\left(t\right)$ are shown in Figure 7b.

**Figure 8:**
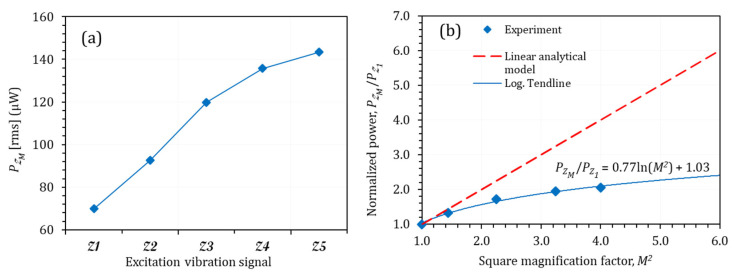
(**a**) Output power for different vibration-level multi-frequency excitation signals and (**b**) normalised power versus magnification factor.

The optimum power with 21.8 kΩ for different magnification factors is shown in Figure 8a. As expected, the power increased with the increase in the excitation vibration level; however, a nonlinear variation was observed. Further analyses were conducted on dimensionless experimental power versus square magnification factor (Figure 8b). Figure 8b shows that the experimental correlation between P and $M^{2}$ was not linear, while according to the analytical modelling of P∝$M^{2}$, the empirical correlation between power and the magnification factor is given by

(11) $$\frac{P_{Z_{M}}}{P_{Z_{1}}}=0.77\ln M^{2}$$

The output power obtained in the experiments was smaller than that obtained by employing the linear analytical theory. For explaining this statement, the interaction effects between the harmonics needed to be studied.

### 5.4. Interaction Effects between Different Excitation Harmonics 

The interaction effects were studied by applying an excitation signal comprising six harmonics and controlling the harmonic amplitude. The excitation signal is denoted by

(12) $$Z\left(t\right)=\alpha_{1}sin\left(\Omega_{1}t\right)+\alpha_{2}sin\left(\Omega_{2}t\right)+\alpha_{3}sin\left(\Omega_{3}t\right)+\alpha_{4}sin\left(\Omega_{4}t\right)+\alpha_{5}sin\left(\Omega_{5}t\right)+\alpha_{6}sin\left(\Omega_{6}t\right)$$

where $\alpha_{1}$ *to* $\alpha_{6}$ can be controllably changed. Note that $\Omega_{i}=i\times \omega_{r=1}$, i=1, …, 6.

A three-level experimental design was proposed so that nonlinear relationships could also be captured and the overlap of the interaction effects could be avoided. In addition, to avoid overlapping the interaction effects, two levels for $\alpha_{1}$ and three levels for $\alpha_{2}$ *to* $\alpha_{6}$ were employed. A fractional factorial design using the orthogonal method of resolution III was used with 36 runs of experiments [32]. Table 4 shows the orthogonal array with thirty-six runs. Moreover, three duplications were performed.

Table 5 shows the Analysis of Variance (ANOVA) table with which the meaningful variations were interpreted. Parameter F is the mean square of responses within the same treatment factor divided by the mean square of responses in all experimental runs. F>1 indicates meaningful variation, and a greater F demonstrates a more sensitive treatment factor. The ANOVA table shows that variables $\alpha_{1}$ to $\alpha_{4}$ have F>1, meaning that their influence on the output power was substantial. Table 5 shows that the influence of the $\alpha_{1}$ factor (with $\Omega_{1}$ frequency) was larger than that of the other factors and that the excitation frequencies of $\Omega_{1}$ to $\Omega_{4}$ had meaningful effects on the output power. This conclusion implies that harmonics in a practical vibration source up to the fourth harmonic should be considered.

**Figure 9:**
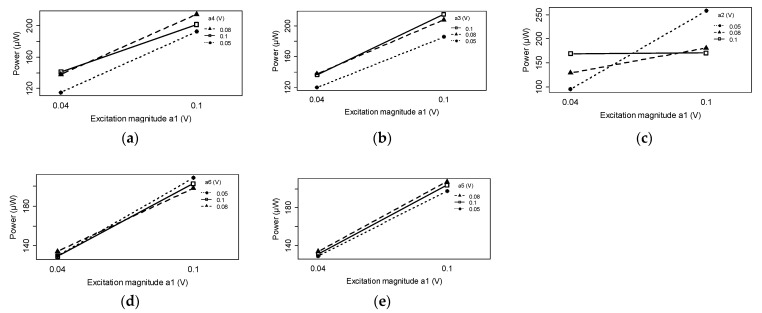
Interaction effects of different excitation harmonics in a multi-frequency excitation signal: (**a**) $\Omega_{1}$–$\Omega_{4}$ effect; (**b**) $\Omega_{1}$–$\Omega_{3}$ effect; (**c**) $\Omega_{1}$–$\Omega_{2}$ effect; (**d**) $\Omega_{1}$–$\Omega_{6}$ effect; (**e**) $\Omega_{1}$–$\Omega_{5}$ effect.

Figure 9a–e demonstrate the interaction effects of different harmonics. The interaction effects of the $\Omega_{1}–\Omega_{2}$, $\Omega_{1}–\Omega_{3}$, and $\Omega_{1}–\Omega_{4}$ harmonics were substantial, while the $\Omega_{5}$ and $\Omega_{6}$ harmonics had negligible effects. The charge cancellation effect of the $\Omega_{1}–\Omega_{2}$ harmonics was visible, i.e., increasing $\alpha_{2}$ (with $\Omega_{2}$ frequency) adversely affected $\alpha_{1}$ (with $\Omega_{1}$ frequency) and increased the effect on power generation. In $\Omega_{1}–\Omega_{3}$ and $\Omega_{1}–\Omega_{4}$ harmonics, the interaction effects were positive. In other words, increasing $\alpha_{3}$ (with $\Omega_{3}$ frequency) or $\alpha_{4}$ (with $\Omega_{4}$ frequency) did not change the positive effect of the first harmonic.

The interaction study showed that the negative interaction between the $\Omega_{1}$ harmonic and the $\Omega_{2}$ harmonic led to smaller power generation than when applying the linear theory, which is observed in Figure 8b. This observation can be linked to the link between the resonant harvester frequencies and the different harmonics in the excitation signal. As demonstrated in Table 6, the first resonant frequency and the piezoelectric-coupled resonant frequency ($\omega_{r=1}$ and $\omega_{\mathrm{pc}}$) were in the range of the first and second excitation harmonics ($\Omega_{i=1}$ and $\Omega_{i=2}$). Thus, these two excitation harmonics are expected to have considerable effects compared with the effects of high harmonics.

### 5.5. Proposing a Nonlinear Model

Large-deformation strain and nonlinear stress–strain constitutive equations are proposed.

Base excitation a(t) deforms the piezoelectric beam with the deformation shape of w(x,t). In a nonlinear framework, the axial strain is given by

(13) $$\varepsilon_{xx}=-z\frac{\frac{\partial^{2}w\left(x,t\right)}{\partial x^{2}}}{{\left(1+{\left(\frac{\partial w\left(x,t\right)}{\partial x}\right)}^{2}\right)}^{\frac{3}{2}}}$$

at level z from the neutral axis.

Moreover, the nonlinear stress–strain constitutive equations can be given by [33]

(14) $$T_{xx}^{p}=Y_{p}\varepsilon_{xx}-{\bar{e}}_{31}E_{z}+\frac{1}{2}\beta_{p}\varepsilon_{xx}^{2}$$

(15) $$T_{xx}^{s}=Y_{s}\varepsilon_{xx}+\frac{1}{2}\beta_{s}\varepsilon_{xx}^{2}$$

where $T_{xx}$ is the axial stress, $E_{z}$ is the z-component electrical field, and β is the nonlinear coefficient.

Thus, the internal bending moment can be calculated with

(16) $$M\left(x,t\right)=-b\left(\int_{-Z_{a}}^{-Z_{b}}T_{xx}^{s}zdz+\int_{-Z_{b}}^{0}T_{xx}^{p}zdz+\int_{0}^{Z_{c}}T_{xx}^{p}zdz\right)$$

Consequently, the beam stiffness (YI) and piezoelectric coupling coefficients ($\Upsilon_{r}$ and $\Lambda_{r}$) are obtained for the nonlinear model.

The electromechanical voltage equation for the nonlinear model is given by

(17) $$C_{P}{\dot{V}}_{R}\left(t\right)+\frac{1}{R}V_{R}\left(t\right)=-\frac{{\bar{e}}_{31}\left(h_{p}+h_{s}\right)b}{2}\overset{L}{\underset{0}{\int }}\frac{d}{dt}\left[\frac{\partial^{2}w\left(x,t\right)}{\partial x^{2}}\left(1+{\left(\frac{\partial w\left(x,t\right)}{\partial x}\right)}^{2}\right)\right]dx$$

More details about the nonlinear model can be found in ref. [34].

## 6. Concluding Remarks and Future Works

This paper presents extensive experimental works aimed at practical PEH performance characterisation. Single-frequency harmonics with resonant and off-resonant excitation frequencies, and multi-frequency signals as practical vibration signals were studied as PVEH excitation signals. The investigations demonstrated the high-strain-level nonlinearities at resonance, while off-resonant harmonics followed the linear theories. Furthermore, with multi-frequency signals, resonance nonlinearity and different frequency interactions caused nonlinear performance in the PEH. This paper contributes to the realistic evaluation of piezoelectric energy harvesters. Nonlinear energy harvester models with more experimental investigation are proposed for future work. Moreover, further studies on the parameters of the flexibility characteristics of energy harvesters, such as thickness and width, are needed.

## Figures and Tables

**Table 1:** Parameters of the unimorph harvesting model.

	Definition	Formula	Parameter	
$\omega_{r}$	Natural frequency	${\left(\lambda_{r}L\right)}^{2}\sqrt{\frac{YI}{m^{*}L^{4}}}$	L	Beam length (m)
			b	Beam width (m)
YI	Beam stiffness b3[Ys(Zb3−Za3)+Yp(Zc3−Zb3)]		h	Layer thickness (m)
			ρ	Density ($kg/m^{3})$
$m^{*}$	Mass per unit length $b\left(\rho_{p}h_{p}+\rho_{s}h_{s}\right)$		Y	Elastic modulus (Pa)
$\lambda_{r}$	Natural frequency coefficient, 1.875, 4.694, 7.885		$\zeta_{r}$	Damping coefficient
*Y_r_*	Piezoelectric energy conversion modal coefficient	$𝒫\left({\frac{d\phi_{r}\left(x\right)}{dx}|}_{x=L}\right)$	${\bar{\varepsilon }}_{33}$	Permittivity (*F*/*M*)
			R	Electrical load (Ω)
𝒫	$-\frac{{\bar{e}}_{31}b}{2h_{p}}\left[Z_{c}^{2}-Z_{b}^{2}\right]$		$Z_{a}$	Z-distance of neutral axis
$\Lambda_{r}$	Piezoelectric reverse energy conversion modal coefficient	$-\frac{{\bar{e}}_{31}\left(h_{p}+h_{s}\right)b}{2}\left({\frac{d\phi \left(x\right)}{dx}|}_{x=L}\right)$	$Z_{b}$	Z-distance: piezo-bottom to neutral axis
			$Z_{c}$	Z-distance: neutral axis to the top
$C_{P}$	Piezoelectric capacitance	$\frac{{\bar{\varepsilon }}_{33}bL}{h_{p}}$	Subscript p	Piezoelectric layer
$\phi_{r}\left(x\right)$	Harvesting beam mode shapes	$\chi_{r}\left[cosh\left(\lambda_{r}x\right)-cos\left(\lambda_{r}x\right)+\alpha_{i}\left(sinh\left(\lambda_{r}x\right)-sin\left(\lambda_{r}x\right)\right)\right]$	Subscript s	Substrate layer

**Table 2:** Comparison of undamped natural frequencies between the presented method and the experiment.

	Undamped Natural Frequencies *ω_r_* (Hz)		
	Experiment	Current Method (Presented in Section 2)	Error (Hz)
First bending mode	20.4	21.7	1.3 Hz
Second bending mode	―	136.3	―
Third bending mode	―	381.5	―
Fourth bending mode	―	747.7	―
Fifth bending mode	―	1235.9	―

**Table 3:** Base excitation signal characteristics and experimental results obtained by connecting harvester to optimum load.

$\Omega_{i}$	$Y_{J}$ (V)	$P_{rms}\left(\mu W\right)$	Experimental Error (%)	$\Omega_{i}$	$Y_{J}$ (V)	$P_{rms}\left(\mu W\right)$	Experimental Error (%)
$\Omega_{1}=1\times \omega_{r=1}=20.4\;\mathrm{Hz}$	0.04	19.51	1.22	$\Omega_{3}=3\times \omega_{r=1}=61.2\;\mathrm{Hz}$	0.04	4.01	1.13
	0.05	38.25	1.25		0.05	6.36	0.33
	0.075	140.68	1.20		0.075	13.73	0.55
	0.09	243.13	1.54		0.09	19.45	0.49
	0.1	344.21	1.18		0.1	24.08	1.71
	0.12	543.71	0.29	$\Omega_{4}=4\times \omega_{r=1}=81.6\;\mathrm{Hz}$	0.04	3.01	2.75
	0.17	1104.92	0.29		0.05	4.87	0.78
	0.2	1370.04	1.70		0.075	10.13	0.46
$\Omega_{2}=2\times \omega_{r=1}=40.8\;\mathrm{Hz}$	0.04	6.98	1.76		0.09	14.32	0.81
	0.05	11.45	1.05		0.1	17.44	0.47
	0.075	24.91	0.75	$\Omega_{5}=5\times \omega_{r=1}=102.0\;\mathrm{Hz}$	0.05	4.34	3.26
	0.09	35	0.82		0.08	10.65	1.78
	0.1	43.45	0.58		0.1	17.25	3.85
				$\Omega_{6}=6\times \omega_{r=1}=122.4\;\mathrm{Hz}$	0.05	3.84	2.32
					0.08	9.2	0.96
					0.1	14.12	2.99

**Table 4:** Orthogonal design of experiments on interaction effects of harmonics.

Index	$\alpha_{1}\;(V)$	$\alpha_{2}\;(V)$	$\alpha_{3}\;(V)$	$\alpha_{4}\;(V)$	$\alpha_{5}\;(V)$	$\alpha_{6}\;(V)$	Index	$\alpha_{1}(V)$	$\alpha_{2}\;(V)$	$\alpha_{3}\;(V)$	$\alpha_{4}\;(V)$	$\alpha_{5}\;(V)$	$\alpha_{6}\;(V)$
1	0.04	0.05	0.05	0.05	0.05	0.05	19	0.1	0.05	0.05	0.08	0.05	0.08
2	0.04	0.08	0.08	0.08	0.08	0.08	20	0.1	0.08	0.08	0.1	0.08	0.1
3	0.04	0.1	0.1	0.1	0.1	0.1	21	0.1	0.1	0.1	0.05	0.1	0.05
4	0.04	0.05	0.05	0.08	0.08	0.1	22	0.1	0.05	0.05	0.1	0.1	0.1
5	0.04	0.08	0.08	0.1	0.1	0.05	23	0.1	0.08	0.08	0.05	0.05	0.05
6	0.04	0.1	0.1	0.05	0.05	0.08	24	0.1	0.1	0.1	0.08	0.08	0.08
7	0.04	0.05	0.08	0.05	0.1	0.1	25	0.1	0.05	0.1	0.08	0.05	0.1
8	0.04	0.08	0.1	0.08	0.05	0.05	26	0.1	0.08	0.05	0.1	0.08	0.05
9	0.04	0.1	0.05	0.1	0.08	0.08	27	0.1	0.1	0.08	0.05	0.1	0.08
10	0.04	0.05	0.08	0.1	0.05	0.08	28	0.1	0.05	0.1	0.1	0.08	0.05
11	0.04	0.08	0.1	0.05	0.08	0.1	29	0.1	0.08	0.05	0.05	0.1	0.08
12	0.04	0.1	0.05	0.08	0.1	0.05	30	0.1	0.1	0.08	0.08	0.05	0.1
13	0.04	0.05	0.1	0.05	0.08	0.05	31	0.1	0.05	0.08	0.05	0.08	0.08
14	0.04	0.08	0.05	0.08	0.1	0.08	32	0.1	0.08	0.1	0.08	0.1	0.1
15	0.04	0.1	0.08	0.1	0.05	0.1	33	0.1	0.1	0.05	0.1	0.05	0.05
16	0.04	0.05	0.1	0.1	0.1	0.08	34	0.1	0.05	0.08	0.08	0.1	0.05
17	0.04	0.08	0.05	0.05	0.05	0.1	35	0.1	0.08	0.1	0.1	0.05	0.08
18	0.04	0.1	0.08	0.08	0.08	0.05	36	0.1	0.1	0.05	0.05	0.08	0.1

**Table 5:** Analysis of Variance (ANOVA) table of the designed test with three duplications.

Variable	$\mathrm{Standard}\;\mathrm{Deviation}\;\mathrm{of}\;{\bar{P}}_{R}$	Degree of Freedom	Mean Square	F
$\alpha_{1}$$\;(\mathrm{with}\;\Omega_{1}$ frequency)	$\sigma_{G_{1}}$	1	$\sigma_{G_{1}}$ = 138,434.3	$\sigma_{G_{1}}/\left(\sigma_{e}/96\right)$ = 109.59
$\alpha_{2}$$\;(\mathrm{with}\;\Omega_{2}$ frequency)	$\sigma_{G_{2}}$	2	$\sigma_{G_{2}}/2$ = 4418.2	$\left(\sigma_{G_{2}}/2\right)/\left(\sigma_{e}/96\right)$ = 3.50
$\alpha_{3}$$\;(\mathrm{with}\;\Omega_{3}$ frequency)	$\sigma_{G_{3}}$	2	$\sigma_{G_{3}}/2$ = 5538.2	$\left(\sigma_{G_{3}}/2\right)/\left(\sigma_{e}/96\right)$ = 4.38
$\alpha_{4}$$\;(\mathrm{with}\;\Omega_{4}$ frequency)	$\sigma_{G_{4}}$	2	$\sigma_{G_{4}}/2$ = 4964.7	$\left(\sigma_{G_{4}}/2\right)/\left(\sigma_{e}/96\right)$ = 3.93
$\alpha_{5}$$\;(\mathrm{with}\;\Omega_{5}$ frequency)	$\sigma_{G_{5}}$	2	$\sigma_{G_{5}}/2$ = 507.4	$\left(\sigma_{G_{5}}/2\right)/\left(\sigma_{e}/96\right)$ = 0.40
$\alpha_{6}$$\;(\mathrm{with}\;\Omega_{6}$ frequency)	$\sigma_{G_{6}}$	2	$\sigma_{G_{6}}/2$ = 119.5	$\left(\sigma_{G_{6}}/2\right)/\left(\sigma_{e}/96\right)$ = 0.09
Residual	$\sigma_{e}$	$3\times 2^{5}$ = 96	$\sigma_{e}/96$ = 1263.2	

**Table 6:** Comparison of the harvester’s resonant frequencies and the external excitation harmonic frequencies.

Harvester’s Resonant Frequencies	External Excitation Frequencies
$\omega_{r=1}$ **= 21.7 Hz**	$\Omega_{i=1}$ **= 20.4 Hz**
$\omega_{pc}$ **= 31.0 Hz**	$\Omega_{i=2}$ **= 40.8 Hz**
$\omega_{r=2}$ = 136.3 Hz	$\Omega_{i=3}$ = 61.2 Hz
$\omega_{r=3}$ = 381.5 Hz	$\Omega_{i=4}$ = 81.6 Hz
$\omega_{r=4}$ = 747.7 Hz	$\Omega_{i=5}$ = 102.0 Hz
$\omega_{r=5}$ = 1235.9 Hz	$\Omega_{i=6}$ = 124.4 Hz

## Data Availability

Not applicable.
